# Hüftfrakturinzidenz und Lockdown: Gibt es Zusammenhänge?

**DOI:** 10.1007/s41970-022-00179-9

**Published:** 2022-03-28

**Authors:** Oliver Malle, Hans Peter Dimai

**Affiliations:** Universitätsklinik für Innere Medizin, Abteilung für Endokrinologie und Diabetologie, Medizinische Universität Graz, Auenbruggerplatz 15, 8036 Graz, Österreich

**Keywords:** Osteoporose, COVID-19, Fraktur, Frakturhäufigkeit, Isolation, Osteoporosis, COVID-19, Fracture, Fracture cases, Isolation

## Abstract

Zur Eindämmung der COVID-19-Pandemie wurde in Österreich bereits mehrfach ein Lockdown verhängt. Durch die häusliche Isolation und dadurch reduzierte physische Aktivität könnte die Frakturhäufigkeit initial positiv beeinflusst werden, aber mittel- bis langfristig aufgrund eines reduzierten Trainingsstatus und verringerter Koordinationsfähigkeit, welche zu einem erhöhten Sturz- und damit Frakturrisiko führen, steigen. Basierend auf Daten der österreichischen Allgemeinen Unfallversicherungsanstalt (AUVA) zeigte sich die Häufigkeit von Hüftfrakturen im Zeitraum des ersten Lockdowns (16. März bis 31. Mai 2020) unverändert im Vergleich zu den gleichen Zeiträumen der Jahre zuvor, jedoch ergab eine Subanalyse eine reduzierte Frakturrate bei Frauen in der Altersgruppe 50-69 Jahre während des ersten Lockdowns verglichen mit dem gleichen Zeitraum des Jahres 2018.

## Abstract (en)

Social lockdown has been repeatedly imposed worldwide due to the coronavirus disease 2019 (COVID‐19). Resultant isolation with less physical activity and restricted health care access may have an impact on fracture incidences. Based on the data of the Austrian Workers’ Compensation Board (AUVA), which is the social insurance for the majority in Austria, hip fracture cases did not differ in the time period March 16th to May 31st 2020 (first lockdown period in Austria) compared to the same period of previous years (2016–2019). Stratified by sex and age group, a lower fracture rate was seen in women aged 50–69 years (*p* < 0.05) in 2020 compared to 2018.

Um eine drohende Überlastung des Gesundheitssystems durch die COVID-19-Pandemie zu verhindern, musste die österreichische Regierung inzwischen schon mehrmalig einen Lockdown verhängen. Nachdem der erste Fall in Europa am 25. Januar 2020 beschrieben worden war und die Weltgesundheitsorganisation (WHO) am 11. März 2020 den COVID-19-Ausbruch zur Pandemie erklärt hatte, wurde der erste Lockdown in Österreich am 16. März 2020 beschlossen und damit das öffentliche Leben komplett heruntergefahren. Diese Maßnahme blieb in vollem Ausmaß bis zum 31. Mai 2020 bestehen [1, 2]. Durch solche drastischen Maßnahmen sind Effekte auf das Gesundheitsverhalten zu erwarten. So konnten bereits zahlreiche negative Veränderungen gezeigt werden, die aus einer häuslichen Isolation resultieren, wie etwa ein Bewegungsmangel, ungesündere Ernährungsweise und Gewichtszunahme sowie vermehrter Tabak- und Alkoholkonsum [3–7].

Aus osteologischer Sicht sind sowohl positive als auch negative Auswirkungen als Folge einer Quarantänemaßnahme denkbar. Eine Isolation führt zwangsläufig zu reduzierter physischer Aktivität und in Folge zu einem reduzierten Trainingsstatus, welcher negative Auswirkungen auf die Koordination hat und damit eine erhöhte Sturzgefahr bzw. erhöhtes Frakturrisiko vermuten lässt. Ebenso nachvollziehbar wäre eine erhöhte Frakturgefahr durch vermehrte körperliche Betätigung nach Aufhebung eines Lockdowns. Des Weiteren konnte gezeigt werden, dass der Schwäche- und Erschöpfungszustand im Rahmen einer COVID-19-Erkrankung selbst zu einer erhöhten Sturzneigung führt [8]. Sogar bei asymptomatischen COVID-19-Erkrankten fand sich ein erhöhtes Sturzrisiko [9].

Betrachtet man Ergebnisse internationaler Studien, die den Einfluss eines Lockdowns auf die Frakturrate untersuchten, findet man heterogene Ergebnisse. Während Studien aus dem Vereinigten Königreich, die Fallzahlen im Zeitraum des Lockdowns mit dem Vorjahr verglichen, eine unveränderte Rate an Frakturen zeigten [10], fanden französische Studien, welche ebenso Vergleiche mit dem Vorjahr herstellten, eine signifikante Reduktion von Hüftfrakturen um 11 % [11]. Letztere verifizierten interessanterweise die stärkste Reduktion traumatologischer Vorstellungen im Bereich der oberen Extremität (13,1 % vs. 30,8 %, *p*  0,0001), wobei der Anteil häuslicher Unfälle erwartungsgemäß massiv anstieg (66,5 % vs. 32,3 %, *p*  0,0001) [12]. Auch eine Studie aus Italien stellte eine signifikante Reduktion der Frakturrate im Vergleich zum Jahr zuvor fest [13].

Es wurde vermutet, dass aufgrund der Angst vor einem damals noch ziemlich unbekannten neuen Virus und der erhöhten Infektionsgefahr in Gesundheitseinrichtungen viele Menschen zögerten, ein Krankenhaus oder eine Notaufnahme aufzusuchen. Ambulanzen mehrerer Bereiche verifizierten im Zeitraum des Lockdowns ein signifikant reduziertes PatientInnenaufkommen, wobei diese Reduktion am deutlichsten in orthopädisch-traumatologischen Institutionen zu finden war [7]. Ferner konnte gezeigt werden, dass PatientInnen, die mit einer Fraktur in einer orthopädisch-traumatologischen Ambulanz vorstellig wurden und durch das Screening positiv auf SARS-CoV‑2 getestet wurden, eine erhöhte Mortalität aufwiesen [14, 15]. Bedauerlicherweise zeigten Daten auch bei negativem SARS-CoV-2-Ergebnis im Zeitraum des Lockdowns eine erhöhte Mortalität [9, 16], sodass neben der Viruserkrankung per se weitere Faktoren mit negativem Einfluss auf das Outcome eine Rolle spielen müssen, wobei man in der Literatur erneut widersprüchliche Ergebnisse findet. Während manche keine Veränderung der Versorgungs- und Behandlungsqualität von Frakturen beschrieben [10, 17], wiesen andere eine verlängerte Wartezeit zur operativen Versorgung nach [16]. Aufgrund der Forcierung einer möglichst frühzeitigen Entlassung sowohl von Arzt/Ärztin als auch PatientIn fand sich im Großteil der Studien eine signifikant reduzierte Krankenaufenthaltsdauer [7, 10].

Dieser Artikel beschreibt im Folgenden eine Analyse, welche die Situation in Österreich beschreibt und auf Daten der österreichischen Allgemeinen Unfallversicherungsanstalt (AUVA) beruht, welcher rund 4,5 Mio. Personen anvertraut sind und damit gut die österreichische Bevölkerung repräsentiert. Im Gegensatz zu den meisten internationalen Studien, welche Vergleiche nur mit dem Vorjahr herstellten, beinhaltet diese Analyse Daten der Hüftfrakturinzidenz bis zum Jahr 2016. Während die mittlere Hüftfrakturhäufigkeit im Zeitraum des ersten Lockdowns (16. März bis 31. Mai 2020) in Österreich durchschnittlich 438 in den Vorjahren betragen hatte, so zeigte sich eine statistisch unveränderte Rate mit 445 im selben Zeitraum des Jahres 2020. Ebenso fand sich bei geschlechtergetrennter Analyse kein signifikanter Unterschied. Auch bei Betrachtung wöchentlicher Zeitintervalle zeigten sich die Frakturraten im Jahr 2020 vergleichbar mit den Jahren zuvor (Abb. 1). Nach Aufteilung in Altersgruppen fand sich jedoch im Jahr 2020 eine signifikant reduzierte Hüftfrakturrate in der Altersgruppe 50–69 Jahre. Eine Analyse der wöchentlichen Zeitintervalle verifizierte, dass diese erniedrigte Frakturrate jedoch nur im Mittelwertvergleich mit dem Jahr 2018 statistisch signifikant war. Zudem konnte diese signifikant reduzierte Rate nur bei weiblichen Individuen festgestellt werden (Tab. 1). Zusammenfassend kann also festgehalten werden, dass sich im Zeitraum des ersten Lockdowns in Österreich im Vergleich zu den Vorjahren bei weiblichen Individuen in der Altersgruppe 50–69 Jahre eine reduzierte Hüftfrakturrate, ansonsten aber keine signifikante Änderung zu finden war.

**Figure 1:**
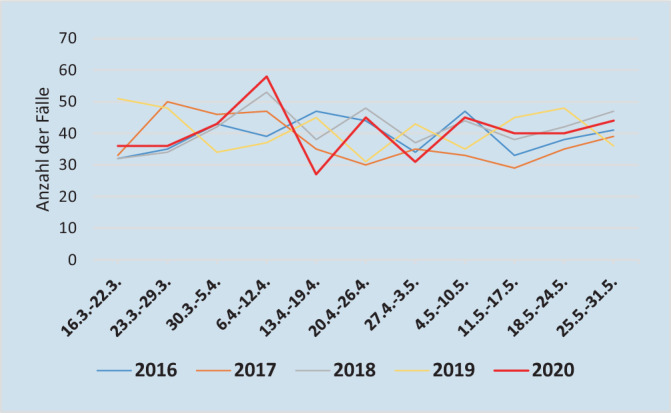

**Table 1:** 

Frakturraten von Frauen 50–69 Jahre	2016	2017	2018	2019	2020
*16.03.–22.03.*	4	5	2	1	2
*23.03.–29.03.*	1	5	4	7	0
*30.03.–05.04.*	6	4	4	0	6
*06.04.–12.04.*	4	2	8	5	5
*13.04.–19.04.*	0	3	4	6	1
*20.04.–26.04.*	2	1	6	7	2
*27.04.–03.05.*	5	3	2	5	1
*04.05.–10.05.*	5	2	7	3	0
*11.05.–17.05.*	0	3	6	4	2
*18.05.–24.05.*	4	3	5	5	5
*25.05.–31.05.*	1	1	6	1	2
Mittelwert (± SD)	3,0 ± 2,2	2,9 ± 1,4	4,9 ± 1,9	4,0 ± 2,4	2,4 ± 2,1
*p*-Wert (t-Test vs. 2020)	0,44	0,49	**0,01**	0,17	–

Diese österreichischen Daten gliedern sich in die bestehende Heterogenität internationaler Analysen ein. Durch die Quarantäne zwangsläufig reduzierte Outdooraktivitäten und sportliche Betätigungen könnten die gezeigte Tendenz zur Reduktion der Frakturrate in der jüngeren Altersgruppe erklären. Da der Großteil der Hüftfrakturen der älteren Generation im häuslichen Bereich auftritt [18], hätte man andererseits bei dieser Population durch die häusliche Isolation eine erhöhte Frakturrate erwarten können, jedoch zeigten sich in den höheren Altersgruppen keine signifikanten Änderungen. Dass die reduzierte Frakturrate nur bei weiblichen Individuen zu finden war, könnte sich durch die niedrige Fallzahl bei ohnehin niedriger Hüftfrakturinzidenz in männlichen Individuen erklären. Vervollständigende Frakturdaten der inzwischen schon mehrmalig verhängten Lockdowns in Österreich sind bald verfügbar und könnten besseren Aufschluss über die Auswirkungen eines Lockdowns auf die Frakturhäufigkeit geben.
